# Comprehensive Urinomic Identification of Protein Alternatives to Creatinine Normalization for Diagnostic Assessment of Lupus Nephritis

**DOI:** 10.3389/fimmu.2022.853778

**Published:** 2022-06-14

**Authors:** Sanam Soomro, Samantha Stanley, Rongwei Lei, Ramesh Saxena, Michelle Petri, Chandra Mohan

**Affiliations:** ^1^ Department of Biomedical Engineering, University of Houston, Houston, TX, United States; ^2^ Department of Internal Medicine, University of Texas Southwestern Medical Center, Dallas, TX, United States; ^3^ Division of Rheumatology, Department of Medicine, Johns Hopkins University, Baltimore, MD, United States

**Keywords:** urine, creatinine, biomarker, SLE, lupus nephritis, point-of-care

## Abstract

**Introduction:**

The current gold standard used for urine biomarker normalization, creatinine, poses a challenge to translate to the point of care because antibodies to creatinine are difficult to develop and currently available ligands to creatinine are sub-optimal for this purpose. Hence, protein alternatives to creatinine are clearly needed. To address this need, lupus nephritis was selected as a model disease where urine protein assessment is required for diagnosis.

**Methods:**

A comprehensive proteomic screen of 1129 proteins in healthy and lupus nephritis urine was executed to identify protein alternatives to creatinine for the normalization of urine biomarkers. Urinary proteins that correlated well with creatinine but did not vary with disease were further validated by ELISA in an independent cohort of lupus nephritis subjects.

**Results:**

The comprehensive proteomic screen identified 14 urine proteins that correlated significantly with urine creatinine but did not differ significantly between SLE and controls. Of the top five proteins selected for ELISA validation, urine HVEM and RELT once again showed significant correlation with urine creatinine in independent cohorts. Normalizing a lupus nephritis biomarker candidate ALCAM using urinary HVEM demonstrated comparable diagnostic ability to creatinine normalization when distinguishing active lupus nephritis from inactive SLE patients.

**Conclusions:**

The discovery of urine HVEM as a protein alternative to creatinine for biomarker normalization has applications in the engineering of antibody-based point of care diagnostics for monitoring lupus nephritis progression.

## Introduction

The advent of personalized medicine and the development of large-scale OMICs technologies have accelerated the discovery of noninvasive biomarkers for diagnostic, prognostic, and therapeutic applications. For diseases affecting the renal system, urine represents a promising body fluid that is potentially enriched for disease biomarkers.

Excessive protein in the urine is an indication of the glomerular filtration barrier becoming compromised, and hence, this is a commonly used marker of renal disease. In addition to assaying total urine protein, specific urine proteins (and metabolites) are interrogated for countless diseases including bladder, prostate, and renal cancer (1–4), drug screening for addiction and therapeutic monitoring, acute kidney injury (5), chronic kidney disease (6), and lupus nephritis (7) and other genitourinary and gynecological conditions. To correctly interpret urine biomarker data, one needs to account for the hydration status of the patient. This is currently achieved by normalizing the biomarker level to urinary creatinine. Creatinine, a waste product of muscle metabolism, is currently the gold standard for urinary glomerular filtration rate normalization (8). Thus, for example, urine albumin creatinine ratio (“ACR”) is a routine diagnostic test for the evaluation of renal diseases, both inflammatory and non-inflammatory in origin.

However, translation of creatinine normalization to point of care diagnostics is a challenge. The most common assay used at the point of care is a sandwich lateral flow assay using antibodies to the target biomarker, best exemplified by the pregnancy test strip employing a sandwich assay for detecting human chorionic gonadotropin (hCG). Translating creatinine normalization to lateral flow point of care assay format has been challenging in that the small size of the creatinine metabolite makes it difficult to generate good antibodies to this molecule, thus limiting the translation of disease-specific urine protein biomarkers to antibody-based point of care applications, including applications in lupus nephritis.

**Figure 1:**
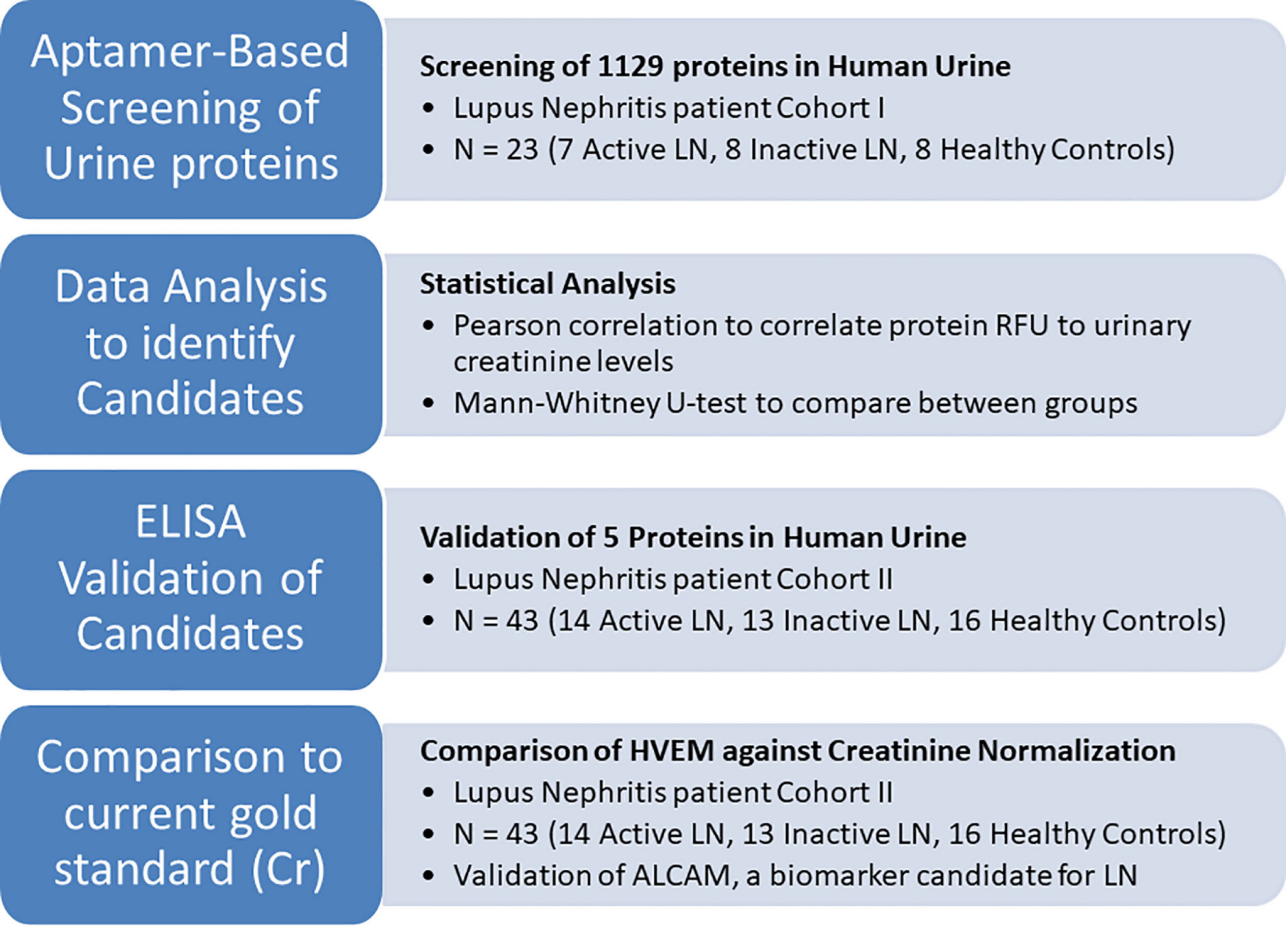
An overview of the current study to identify protein alternatives to creatinine for urine biomarker normalization. First, the urine of 23 human subjects was comprehensively interrogated for 1129 proteins using an aptamer-based proteomic screen. Proteins that correlated well with creatinine and did not differ among patient groups and healthy controls were identified using Pearson correlation and the Mann-Whitney test, respectively. Of these, 5 proteins were chosen for ELISA validation in an independent cohort of 43 subjects. HVEM, the most promising molecule for urine biomarker normalization, was used to normalize the lupus nephritis biomarker candidate urine ALCAM.

To address this bottleneck, we wondered if there could be a protein alternative to creatinine. A proteomic strategy was devised to screen for such molecules, using lupus nephritis as a model of renal disease where proteinuria is common. Indeed, besides the ACR, a multitude or urine proteins are being systematically evaluated to ascertain if they have disease diagnostic potential for this disease. Specifically, a comprehensive proteomic screen of 1129 human proteins, as outlined in **Figure 1**, was undertaken using diseased (lupus nephritis) and healthy urine samples to identify urinary proteins that correlate with urinary creatinine (but not with disease) and can thus be used for normalization of urine biomarker levels. Out of 14 urine proteins that met all selection criteria, five were further validated by ELISA in an independent patient cohort, resulting in the identification of HVEM as the most promising marker for urine biomarker normalization. Having such protein alternatives to urinary creatinine would greatly facilitate urine biomarker monitoring as well as the design of point of care lateral flow tests for routine monitoring of LN, as well other renal diseases.

## Methods

### Human Urine Samples

For the initial aptamer-based targeted proteomic screening, 23 human urine samples were obtained from the University of Texas Southwestern Medical Center (UTSW) consisting of seven active lupus nephritis (LN), eight inactive SLE, and eight healthy controls (HC). Samples were obtained after informed consent at UTSW with Institutional Review Board approvals from UTSW and the University of Houston.

For ELISA validation of the hits from the proteomic screen, 43 human urine samples were obtained from the Johns Hopkins Medical Center (JHMC) and BioreclamationIVT consisting of 14 active LN, 13 inactive disease, and 16 healthy controls. Detailed clinical information pertaining to these subjects is provided in **Table 1**. The patient samples were obtained after informed consent at JHMC with Institutional Review Board approvals from both JHMC and the University of Houston. Active LN patients had biopsy proven LN with the renal component of SLEDAI > 8 (i.e., rSLEDAI > 8), while inactive disease was defined as the rSLEDAI = 0 and SLEDAI < 4. Inactive patients with SLICC (Systemic Lupus Collaborating Clinics) renal activity scores > 4were excluded from the study (9). SLEDAI was determined following the ACR disease guidelines (10). Matched healthy control urine samples were purchased from BioreclamationIVT (Westbury, NY).

**Table 1:** Demographic and clinical characteristic of the primary validation cohort.

Variable	Healthy Controls	Inactive SLE	Active LN
	n=16	n=13	n=14
Race			
Caucasian	7	7	7
African American	9	6	7
Age (yr)			
Mean	40 ± 10.7	48 ± 17.6	39 ± 12.5
Range	27–57	24–70	21–60
SLEDAI			
Mean	N/A	0 ± 0.6	11 ± 2.9
Range	N/A	0–2	8–18
rSLEDAI			
Mean	N/A	0 ± 0	9 ± 1.5
Range	N/A	0–0	8–12

Means are expressed with standard deviation.

NA, Not Available.

### Aptamer-Based Targeted Proteomic Screen of 1129 Proteins

An aptamer-based proteomic screen of 1129 proteins was conducted as described (11). This proteomic platform from Somalogic was used because of its comprehensive coverage (>1000 proteins), high specificity and high sensitivity, allowing for the detection of proteins up to the femtomolar range. The specificity of the assay is derived from the use of a panel of 1129 unique aptamers, which are modified DNA oligonucleotides selected because they were specific to one protein each, with minimal cross-reactivity (12). Urine was diluted 20% in diluent buffer and added to aptamer-coated beads. After incubation for 3.5 hours, the sample was removed and the beads were washed to remove unbound protein. Proteins in the sample that had bound to the aptamer coated beads were then biotinylated. The protein-aptamer complexes were photocleaved, collected, and immobilized on streptavidin coated magnetic beads, where a series of washes ensured specific binding of the aptamers to the proteins. The aptamers were uncoupled from the proteins using a high salt buffer, hybridized onto a DNA microarray, and the results were reported as relative fluorescence units. Proteomic studies were carried out at the Houston Omics Collaborative (https://hoc.bme.uh.edu/).

### Statistical Analysis

The relative fluorescence unit readout from the hybridization array for each aptamer (corresponding to individual protein biomarkers) was normalized across the samples to correct for any variations due to the hybridization procedure, using control samples and probes. R Version 1.0.136 with the readxl, stats (13), and Hmisc, packages were used to carry out further data analysis. Mann-Whitney U-test and Student t-test were used to compare between groups to identify proteins that were significantly different between subject groups. Pearson correlation was used to correlate the relative fluorescent units of each protein in the sample to the urinary creatinine of the subject (Cayman Chemical, Ann Arbor, MI, USA) to identify proteins that correlated well with creatinine.

### ELISA Validation

ELISA validation was carried out for five proteins: Herpesvirus entry mediator (HVEM), bone morphogenetic protein receptor type 2 (BMPRII), Dectin-1, Serine Peptidase Inhibitor Kunitz Type 2 (SPINT2), and Receptor Expressed In Lymphoid Tissues (RELT). Kits were purchased for HVEM (Cat. No. EK1226, Boster Biological Technology, Pleasanton, CA, USA), BMPRII (Cat. No. ELH-BMPR2-1, RayBiotech, Inc., Norcross, GA, USA), Dectin-1 (Cat. No. ELH-DECTIN1-1, RayBiotech, Inc., Norcross, GA, USA), SPINT2 (Cat. No. DY1106, R&D Systems, Inc., Minneapolis, MN, USA), and RELT (Cat. No. SEK10530, Sino Biological Inc., Beijing, China). The samples were also assayed for ALCAM (Cat. No. DY656, R&D Systems, Inc., Minneapolis, MN, USA), a potential urinary biomarker for lupus nephritis (14). Validation data were analyzed and graphed in GraphPad Version 6.05 using the Mann Whitney U-test, receiver operator curves (ROC), and area under the ROC curve (AUC).

## Results

### 1129-Plex Proteomic Screening Results

**Figure 2:**
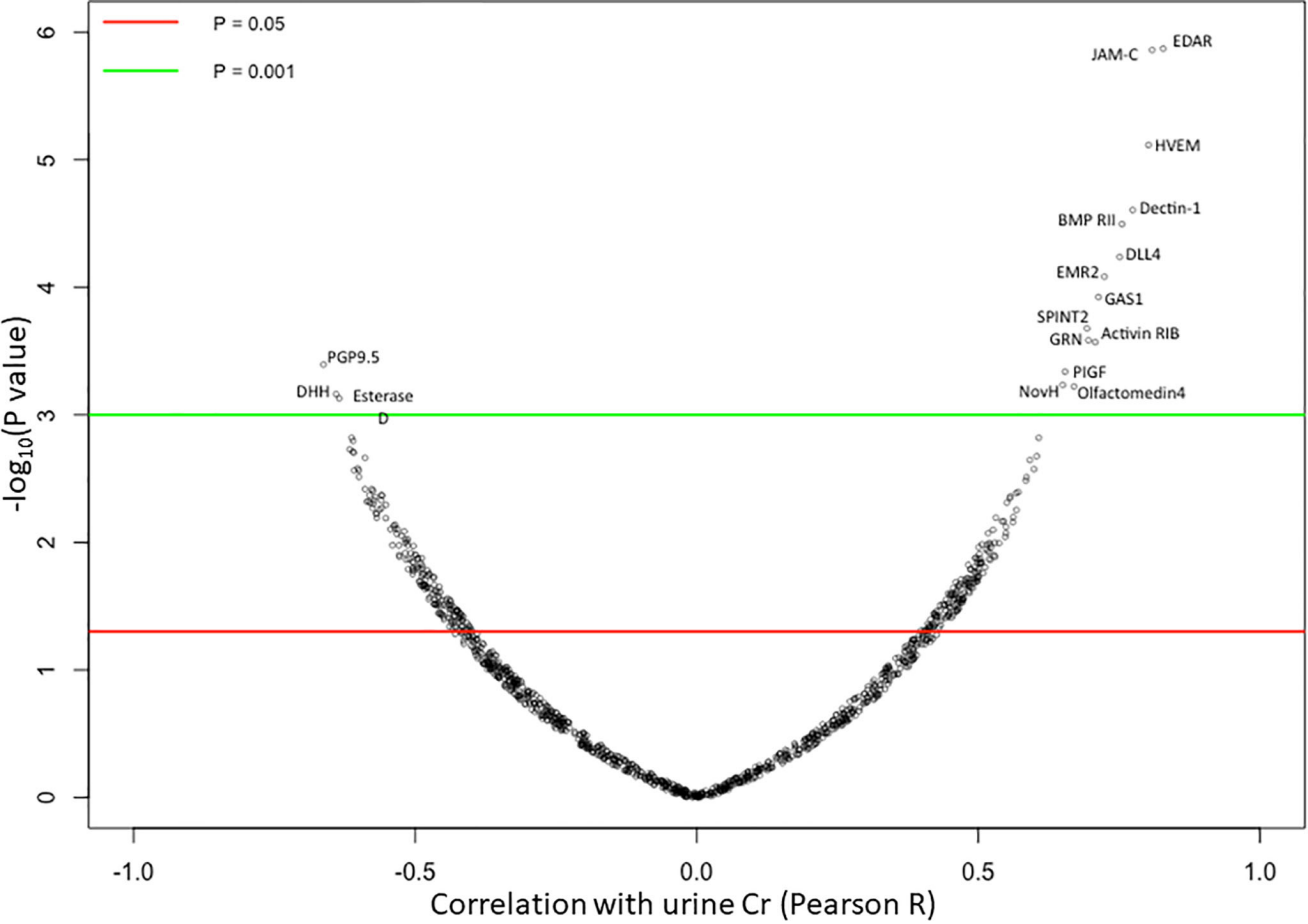
Correlation of 1129 Urine Proteins with Urine Creatinine. Each of the 1129 urine proteins assayed on the aptamer-based screen was correlated to urinary creatinine (using Pearson correlation) in 15 SLE patients and 8 healthy controls, and the results are displayed using a volcano plot. Horizontal red and green lines show threshold P value of the Pearson correlation at P < 0.05 and P < 0.01 respectively. 14 urine proteins were identified to be significantly positively correlated to urine creatinine while 3 urine proteins were identified to be significantly negatively correlated with urine creatinine.

**Figure 3:**
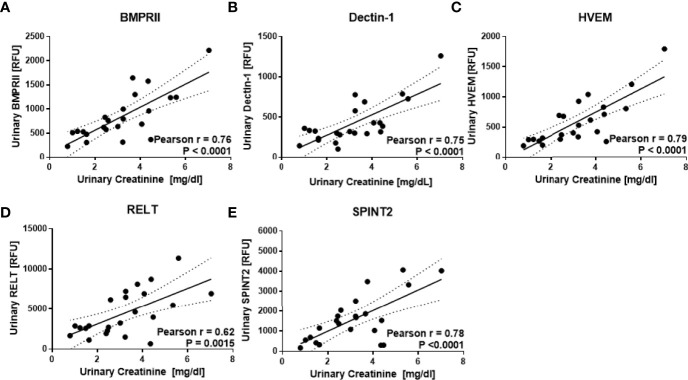
Urine proteins that correlate best with urine creatinine. The aptamer-based screen identified five urine proteins that correlated best with urine creatinine but not differ between subject groups. As described in **Figure 1**, the levels of 1129 urine proteins were assayed in 7 active LN, 8 inactive LN, 8 healthy controls using an aptamer-based proteomic screen. Of these, urine BMPRII, Dectin-1, HVEM, RELT, and SPINT2 **(A–E)** exhibited the highest Pearson R correlation with creatinine but did not differ significantly between the subject groups.

23 human urine samples were screened for the levels of 1129 proteins using a comprehensive targeted proteomic screen. Of the urine proteins interrogated, several were significantly elevated in the urine of patients with active LN (14). As opposed to the previous study that was designed to identify novel urine biomarkers for LN (14), the focus of this study was to identify urine proteins (out of the 1129 interrogated) that correlated best with urine creatinine, and did not vary with disease status. Using Pearson correlation, we identified 62 urine proteins that were positively correlated with creatinine (r > 0.5, P < 0.05), as depicted in **Figure 2**. Of these 62 proteins, 48 were removed from further consideration as they were significantly different between at least two subject groups using Student t-test or Mann Whitney U-test at P < 0.1. Of the remaining 14 proteins, listed in **Table 2**, the top five proteins ranked based on Pearson correlation were HVEM, BMPRII, Dectin-1, SPINT2, and RELT. The correlation of these urine proteins with urine creatinine from the screening assay is summarized in **Figures 3A–E**). These five proteins were chosen for further validation using an orthogonal assay platform (ELISA) in an independent cohort of urine samples.

**Table 2:** Urine proteins that were positively correlated with urinary creatinine but were invariant between LN patients and healthy controls.

UrineProtein	Correlation with Creatinine^†^
HVEM	0.78
BMP RII	0.76
Dectin-1	0.75
SPINT2	0.69
RELT	0.62
CLM6	0.60
JNK2	0.60
PAPP-A	0.58
HSP70 protein 8	0.56
PTN	0.54
Elafin	0.51
IL-1Rrp2	0.51
RASA1	0.50
APP	0.50

^†^Pearson r For this analysis, the indicated urine proteins and creatinine were assayed in 7 active LN, 8 inactive LN, and 8 healthy control urine samples using an aptamer-based screening platform.

### ELISA Validation of Candidate Proteins

**Figure 4:**
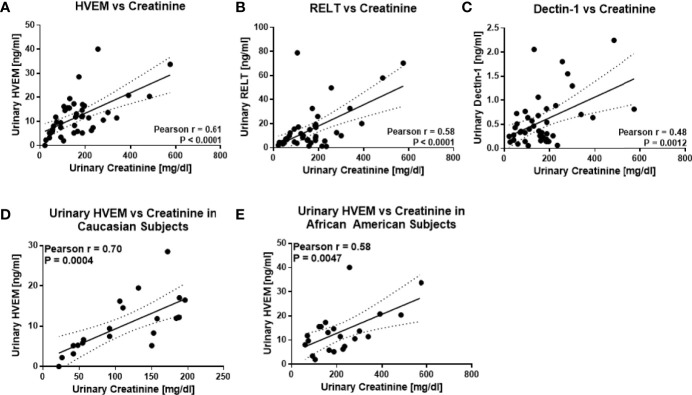
ELISA validation of the top urine protein candidates in an independent cohort of 14 active LN, 13 inactive LN, 16 healthy control subjects. Urine samples were pretested for dilution and assayed for urinary HVEM **(A)**, urinary RELT **(B)**, and urinary Dectin-1 **(C)** in these 43 urine samples. A significant positive correlation was noted between urine HVEM and RELT with urinary creatinine. Urine HVEM, the most promising biomarker for urine biomarker normalization, was assayed in 21 Caucasian **(D)** and 22 African American **(E)** subjects, again showing a significant positive correlation with urine creatinine across both races.

**Figure 5:**
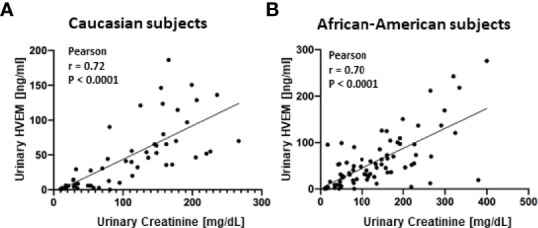
Extended ELISA validation of urine HVEM in an independent cohort of subjects. Urine HVEM, the most promising biomarker for urine biomarker normalization, was assayed in an additional cohort of Caucasian **(A)** and African American **(B)** subjects, again showing a significant positive correlation with urine creatinine across both races. In **(A)**, 23 SLE and 30 healthy urine samples were interrogated. In **(B)**, 49 SLE and 30 healthy urine samples were interrogated.

An independent cohort of 43 urine samples was used for ELISA validation, comprised of 16 HC, 13 inactive SLE, and 14 active LN urine samples. ELISA kits for all five target proteins were pre-tested for their detection sensitivity in urine. Urine BMPRII and SPINT2 were too low in concentration to be detected by ELISA. HVEM, Dectin-1, and RELT were validated further in a total of 43 urine samples. A correlation of these molecules as assayed by ELISA with urinary creatinine is shown in **Figure 4**. Once again, urinary HVEM and RELT were noted to have a significant positive correlation with urinary creatinine (Pearson r = 0.61, P < 0.0001 and r = 0.58, P < 0.0001, respectively) in this independent validation cohort (**Figures 4A, B**). In contrast, urinary Dectin-1 did not show a positive correlation with urinary creatinine in these validation samples (Pearson r = 0.48, P = 0.0012). For the most promising of these proteins, HVEM, the impact of ethnicity was evaluated further. Urinary HVEM correlated with urinary creatinine in both Caucasian and African American subjects (Pearson r = 0.70, P = 0.0004 and Pearson r = 0.58, P = 0.0047, respectively) as shown in **Figures 4D, E**. These findings were extended to an larger independent cohort of lupus patients, again comprised of Caucasian and African American subjects. Again, as shown in **Figures 5A, B**), urinary HVEM correlated with urinary creatinine in both ethnic groups.

### Testing the Ability of Urine HVEM to Normalize Urine Biomarkers in LN

**Figure 6:**
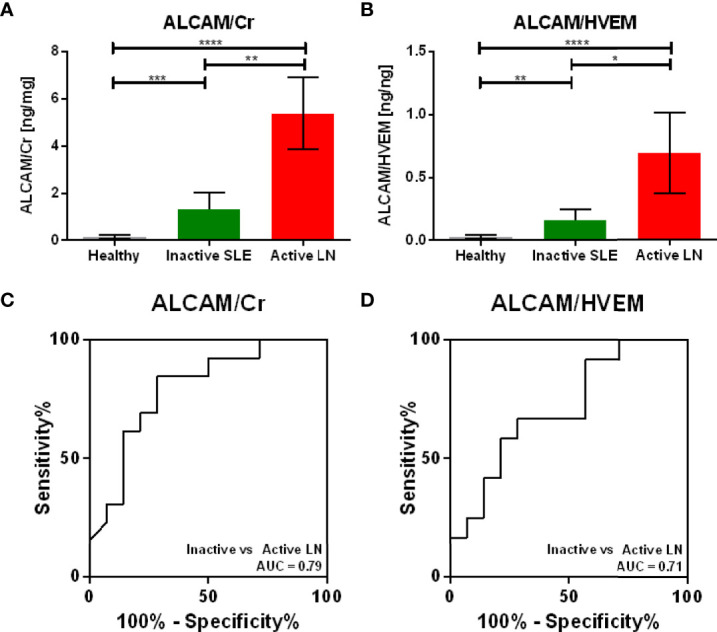
Normalizing Urine ALCAM using Urine HVEM versus Urine Creatinine. As urine HVEM emerged as the most promising marker for urine biomarker normalization, the diagnostic utility of HVEM normalization was compared to the gold standard, creatinine normalization. Normalization of urine ALCAM, a proposed biomarker for diagnosing lupus nephritis, using urinary creatinine **(A)** and urinary HVEM **(B)** shows comparable active versus inactive lupus nephritis ALCAM fold changes of 4.06 and 4.41, respectively. The diagnostic ability of ALCAM normalized with creatinine **(C)** and ALCAM normalized with HVEM **(D)** shows comparable ROC AUC values of 0.79 and 0.71, respectively. Bars show mean ± standard error of the mean. One sample was removed from the plots, as the HVEM concentration was too low to be detected by ELISA. *, **, *** and **** represent p < 0.05, p < 0.01, p < 0.001 and p < 0.0001, respectively.

Given that urinary HVEM correlated consistently with urinary creatinine, we next assessed whether urinary HVEM could be used to normalize urine biomarker levels, just as urinary creatinine is currently used. The same validation cohort of 43 urine samples used above to assay urinary HVEM and creatinine was interrogated for the levels of urinary ALCAM, a biomarker candidate for LN (14). Urine ALCAM normalized by creatinine, the current gold standard, showed a fold change of 4.06 (Mann Whitney U-Test P = 0.0040) between active and inactive lupus nephritis patients, while normalization of urine ALCAM with urine HVEM showed a similar fold change of 4.41 (Mann Whitney U-Test P = 0.0369) between active and inactive lupus nephritis, as shown in **Figures 6A, B**). ROC curves in **Figures 6C, D**) illustrate the diagnostic ability of urine ALCAM for distinguishing active lupus nephritis using the two normalization methods (urine creatinine versus urine HVEM), showing comparable performance characteristics with AUC values of 0.79 and 0.71 respectively.

## Discussion

This study is the first to screen over 1,100 human proteins for markers that could be used to normalize urine biomarkers, using a highly specific and sensitive targeted proteomic platform. By applying statistical criteria and identifying proteins that correlate with creatinine, but not with disease, we have identified 14 urine proteins that could potentially be used for urine biomarker normalization, either in standard laboratory tests, or in point of care applications. This is of practical importance because the metabolite creatinine does not readily lend itself for antibody-based diagnostics.

Urine validation of these candidates by ELISA further supports the need for easily detectable urine markers for biomarker normalization, as two of the five proteins chosen for validation were too low in concentration for ELISA to detect, which will make it even harder to detect these molecules at the point of care. Of the five urinary protein candidates chosen for validation, three, urinary HVEM, Dectin-1, and RELT, were detectable by ELISA. However, urinary Dectin-1 did not correlate with urine creatinine in the larger validation cohort. Hence, urinary HVEM and urinary RELT emerged as the leading urine protein candidates for urine concentration normalization. In this study, urine concentrations of HVEM ranged from 5 ng/ml to 34 ng/ml in healthy subjects and 5 ng/ml to 41 ng/ml in patients with active LN. Urine concentrations of RELT ranged from 1 ng/ml to 71 ng/ml in healthy subjects and 4 ng/ml to 79 ng/ml in patients with active LN. In pursuing urine HVEM further, it exhibited good correlation with urine creatinine in both Caucasian and African American subjects. When using urinary HVEM as a normalization marker for the LN urinary biomarker candidate ALCAM, both HVEM and creatinine normalization exhibited comparable fold changes and ROC AUC values. Unlike creatinine, antibodies to HVEM are readily available, thus rendering it attractive for antibody-based diagnostic assays, of particular relevance in point of care applications.

HVEM is a member of the tumor necrosis factor receptor superfamily and is a cell surface receptor that is used by the herpes simplex virus for cellular entry. It is also involved in the regulation of T-cell responses by inflammatory and inhibitory signaling pathways (15). HVEM is widely expressed in the gallbladder, appendix, lymph nodes, tonsils, spleen, adrenal glands, stomach, rectum, kidney, bladder, and endometrium (16). Expression of HVEM has been documented to be increased in ovarian serous adenocarcinoma tissue (17), colorectal cancer epithelium (18), esophageal squamous cell carcinoma (19), and breast cancer (20). Soluble HVEM has also been implicated in the serum of patients with hepatocellular carcinoma (21), gastric cancer (22), allergic asthma, atopic dermatitis and rheumatoid arthritis (23). HVEM has also been implicated into innate mucosal defense against bacteria by promoting genes associated with immunity in the colon of a mouse model for Escherichia coli infection (24). Interestingly, one report shows that active SLE patients had a significantly higher proportion of circulating HVEM-expressing CD4+T-cells than healthy individuals (25). However, urinary HVEM does not appear to be elevated in renal diseases or in autoimmunity.

This study represents the first comprehensive proteomic screen for urine proteins that can potentially be used as a substitute for urine creatinine, for normalizing urine biomarker levels. We find urine HVEM is not altered in patients with active LN and that urine HVEM correlates well with urine creatinine. The utility of having such a normalizer protein for calibration of urine biomarkers extends beyond lupus nephritis. Urine biomarker testing is widely used for assessing cancers (1–4), multiple renal diseases (5–7), and other diseases (26) as well as for drug testing (27). Even when total protein in urine is assayed in clinical diagnostics, it is normalized to creatinine, in the form of the ACR test. Urinary HVEM can certainly be used for normalization in all of the above scenarios, readily extending these tests to encompass potential point of care assays.

Further studies are warranted in which urinary HVEM and urinary creatinine are compared head-to-head in larger, independent cohorts of lupus nephritis patients as well as in other diseases where urinary biomarkers have diagnostic potential. Renal micropuncture studies are also warranted to detail how HVEM is handled in the nephron to assess if it is neither secreted nor absorbed. Studies are also warranted to assess if HVEM can be used to estimate the glomerular filtration rate. Finally, some of the other urine protein candidates described in this work (e.g., BMPRII, SPINT2) warrant further investigation, with comparisons to urinary creatinine and urinary HVEM.

## Conclusion

The current gold standard used for urine biomarker normalization, creatinine, poses a challenge to translate to point of care applications because antibodies to creatinine are difficult to develop and currently available ligands to creatinine are sub-optimal for this purpose. This comprehensive screen of >1000 proteins has identified urine HVEM as an alternative to creatinine for normalizing the concentrations of urine biomarkers. The discovery of urine HVEM as well as other normalizing proteins paves the way towards accurate monitoring of disease specific urine biomarkers, with unique implications for antibody-based point of care diagnostics.

## Data Availability Statement

The datasets presented in this study can be found in online repositories. The names of the repository/repositories and accession number(s) can be found below: The proteomic profiling data comparing lupus nephritis and controls has previously been published (PMID: 32366845).

## Ethics Statement

The studies involving human participants were reviewed and approved by IRB Boards at University of Houston, John Hopkins Medical University and UT Southwestern Medical Center. The patients/participants provided their written informed consent to participate in this study.

## Author Contributions

SSo and CM designed the study; RS and MP provided the human samples for the study. SSo, SSt, and RL carried out the experiments; SSo analyzed the data; SSo and CM drafted and revised the paper; all authors read and approved the final version of the manuscript.

## Funding

This work is supported by NIH R01 AR074096, George M. O’Brien Kidney Research Core Center (US National Institutes of Health grant P30DK079328) and the Lupus Research Alliance. The Hopkins Lupus Cohort is supported by RO1 AR 69572.

## Conflict of Interest

The authors declare that the research was conducted in the absence of any commercial or financial relationships that could be construed as a potential conflict of interest.

## Publisher’s Note

All claims expressed in this article are solely those of the authors and do not necessarily represent those of their affiliated organizations, or those of the publisher, the editors and the reviewers. Any product that may be evaluated in this article, or claim that may be made by its manufacturer, is not guaranteed or endorsed by the publisher.
